# Enhancement of anti-murine colon cancer immunity by fusion of a SARS fragment to a low-immunogenic carcinoembryonic antigen

**DOI:** 10.1186/1480-9222-14-2

**Published:** 2012-02-03

**Authors:** Chen-Si Lin, Shih-Han Kao, Yu-Cheng Chen, Chi-Han Li, Yuan-Ting Hsieh, Shang-Chih Yang, Chang-Jer Wu, Ru-Ping Lee, Kuang-Wen Liao

**Affiliations:** 1 School of Veterinary Medicine, National Taiwan University, Taipei, Taiwan; 2 Department of Biological Science and Technology, National Chiao-Tung University, Hsin-Chu, Taiwan; 3 Department of Food Science, National Taiwan Ocean University, Keelung, Taiwan; 4 Department of Nursing, Tzu Chi University, Hualien, Taiwan

**Keywords:** immunotherapy, tumor-derived peptide, tumor vaccine, low-immunogenicity

## Abstract

**Background:**

It is widely understood that tumor cells express tumor-associated antigens (TAAs), of which many are usually in low immunogenicity; for example, carcinoembryonic antigen (CEA) is specifically expressed on human colon cancer cells and is viewed as a low-immunogenic TAA. How to activate host immunity against specific TAAs and to suppress tumor growth therefore becomes important in cancer therapy development.

**Results:**

To enhance the immune efficiency of CEA in mice that received, we fused a partial CEA gene with exogenous SARS-CoV fragments. Oral vaccination of an attenuated *Salmonella typhimurium *strain transformed with plasmids encoding CEA-SARS-CoV fusion gene into BALB/c mice elicited significant increases in TNF-α and IL-10 in the serum. In addition, a smaller tumor volume was observed in CT26/CEA-bearing mice who received CEA-SARS-CoV gene therapy in comparison with those administered CEA alone.

**Conclusion:**

The administration of fusing CEA-SARS-CoV fragments may provide a promising strategy for strengthening the anti-tumor efficacy against low-immunogenic endogenous tumor antigens.

## Background

Tumor-associated antigens (TAAs) are attractive candidates for cancer therapy in view of their specificity to tumor targets and safety profile. Although most known tumor antigens can generate cancer-specific immune responses, they are either poorly immunogenic or functionally non-immunogenic, and may not elicit immune responses sufficient to eradicate tumor cells [1]. Therefore, efforts to enhance TAA-mediated host immunity towards tumors are being made by exploring a variety of adjuvants for immunotherapy, adjuvants for cancer vaccines such as highly-immunogenic vectors, combinations of T-cell co-stimulatory molecules, or immune-stimulatory cytokines usually being employed [2]. Carcinoembryonic antigen (CEA) is a well-known and over-expressed TAA in most human colon carcinomas, and vaccination with CEA has been demonstrated to induce, although with only minor effects, both humoral and T-cell responses [3]. In order to magnify the CEA-mediated tumor-specific immune activities, co-administration of CEA with other immune-response enhancers such as granulocyte-macrophage colony-stimulating factor (GM-CSF) [4] or interleukin (IL)-12 [5] has yielded promising results with regards to the activation of cellular and humoral CEA-specific immune responses.

Some antigenic peptides derived from the severe acute respiratory syndrome coronavirus (SARS-CoV) have exhibited strong immunogenicity, and specific immunogenic epitopes (S978 and S1203) of the S protein of SARS-CoV have been demonstrated to exhibit a vigorous affinity towards binding with HLA-A2 and induce high levels of gamma interferon (IFN-γ)-secreting T-cells [6]. Therefore, employment of these highly-immunogenic peptides may boost the positive properties of CEA tumor vaccines.

In this study, we constructed fusion genes by fusing a partial sequence of CEA with highly-immunogenic fragments of SARS-CoV, which have been verified consistently to effectively elicit IFN-γ secretion in humans [6]. For achieving simple administration of drugs and enhancement of host immunity, an oral DNA vaccine vector, *Salmonella typhimurium *(SL3261) [7], was used as a carrier to deliver the SARS-CoV-CEA fusion genes. Using these methods, we sought to demonstrate the administration of a customized and efficient cancer therapy that combines any selected TAAs and immune-activating sequences using a simple drug-delivery method. Significant retardation of tumor growth and increased survival were observed in mice with cancers in our study, and the results obtained using this strategy were therefore promising.

## Results

### Prediction of H2-Kd binding epitopes from CEA-SRAS-CoV fusion polypeptides

**Figure 1:**
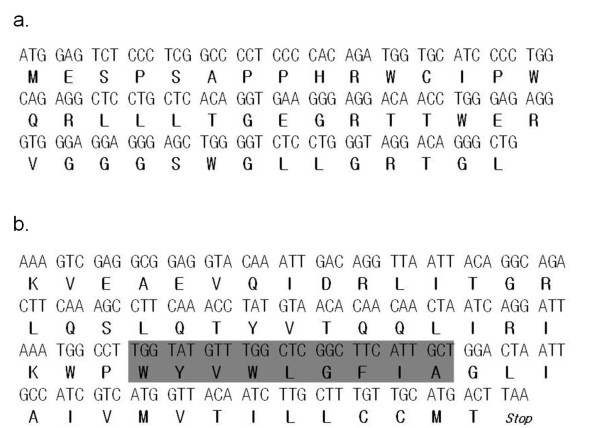
**Flanking nucleotide sequences of the CEA and SARS epitopes**. (a) 132 bps (nucleotides 12804~10935) of the CEA sequence (NCBI accession No. Z21818) and (b) 177 bps of the SARS sequence (NCBI accession No. AB263618) were used as the starting materials to generate highly-immunogenic mutations. The grey area indicates the epitope used for affinity prediction.

The SYFPEITHI prediction algorithm was used to predict peptide/H2-Kd interaction by affinity calculation. To find a low-immunogenic TAA and prevent the human TAA from activating mouse immunity, a truncated CEA peptide was used as a low-immunogenic tumor antigen in this study, the sequence of which is shown in Figure 1a. The CEA sequence was predicted to be less immunogenic by affinity calculation because its highest epitope score (CEA a.a. No. 5-13 (Figure 1a): epitope score 16) was far lower than the scores of well-known epitopes of *Listeria monocytogenes *(Listeriolysin O) (LLO91-99: epitope score 24; p60217-225: epitope score 27) (Table 1). A selected SARS fragment was fused with CEA, and this sequence is shown in Figure 1b. As described previously, the epitope within the CEA-SARS-CoV amino acid sequence was also calculated and compared with known epitopes in *Listeria monocytogenes *(LLO91-99, p60217-225). As a result, the highest score within the CEA-SARS sequence was 21 in the SARS region (Table 2), lower than the scores of known epitopes of *L. monocytogenes *for peptide/H2-Kd interaction. In addition, fusion of the CEA and SARS sequences did not create a new epitope with a higher score (data not shown).

**Table 1:** Comparison of the CEA and CEA-SARS epitopes with other known epitopes that have been proven to elicit immunity in BALB/c mice.

Gene	Primer	Sequence (5'→3')	Tm (°C)
	P1	TACggAATTCATggAgTCTCCCTCggCCCCTCCCCACAgATggTgCATCC	71.6
	P2	CCTggCAgAggCTCCTgCTCACAggTgAAgggAggACAAC	71.5
	P3	CTgggAgAgggTgggAGggAgggAgCTggggTCTCCTgggT	75.1
**CEA**	P4	CTCCTCCCACCCTCTCCCAggTTgTCCTCCCTTCACCTgT	71.8
	P5	gAgCAggAgCCTCTgCCAggggATgCACCATCTgTggggA	73.7
	P6	gCTATC TAgATCACAgCCCTgTCCTACCCAggAgACCCCAgCTCC	71.1
	P7	gCTATCTAgACAgCCCTgTCCTACCCAggAgACCCCAgCTCC	71.0

	P1	TACgTCTAgAAAAgTCgAggCggAggTACAAATTgACAggTTAATTACA	65.7
	P2	ggCAgACTTCAAAgCCTTCAAACCTATgTAACACAACAAC	63.9
	P3	TAATCAggATTAAATggCCTTggTATgTTTggCTCggCTT	65.3
**SARS**	P4	CATTgCTggACTAATTgCCATCgTCATggTTACAATCTTg	63.7
	P5	gCTAAAgCTTTTAAgTCATgCAACAAAgCAAgATTgTAACCATgACgA	64.1
	P6	TggCAATAgTCCAgCAATgAAgCCgAgCCAAACATACCA	67.7
	P7	AggCCATTTAATCCTgATTAgTTgTTgTgTTACATggTT	61.7
	P8	TgAAggCTTTgAAgTCTgCCTgTAATTAACCTGgTCAATTT	63.2

**Table 2:** PCR primers used to synthesize CEA and SARS peptides.

H2-Kd nonamer	Amino acid	Score	References
**CEA**	S**A**PPHRWC**I**	16	NCBI accession No. Z21218
**SARS**	W**Y**VWLGFI**A**	21	NCBI accession No. AB263618
**Listeria LLO_91-99_**	G**Y**KDGNEY**I**	24	Nakamura et al., 2003
**Listeria p60_217-225_**	K**Y**GVSVQD**I**	27	Bouwer et al., 1996
**EGFP_200-208_**	H**Y**LSTQSA**L**	27	Gambotto et al., 2001

Predicted by the SYFPEITHI algorithm.

### Effects of tumor vaccines on cytokine profiles

**Figure 2:**
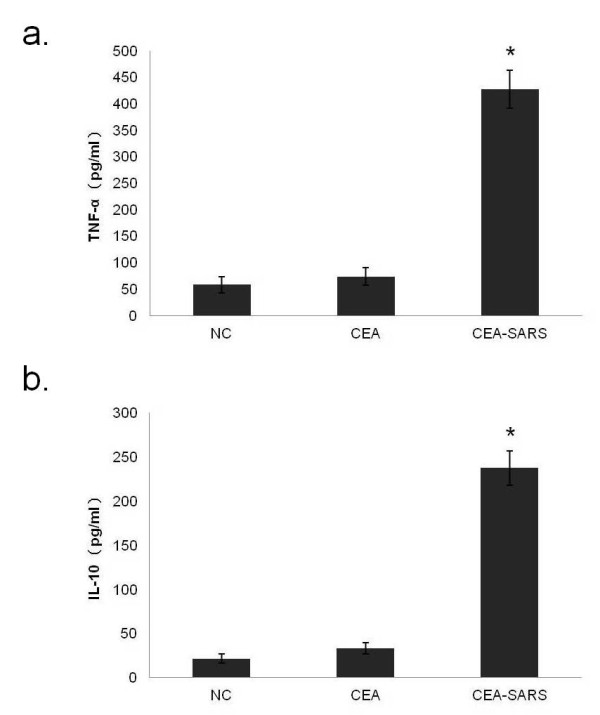
**Serum cytokine profiles of BALB/c mice orally-vaccinated with CEA-SARS-derived epitopes**. Sera samples were collected for *in vivo *cytokine detection, including (a) TNF-α and (b) IL-10. (*, *p *< 0.05 in comparison with the control and the pCEA group).

To examine the cytokine profiles of the different immunized groups quantitatively, sera samples were collected from each group 4 weeks after vaccination (i.e., 2 weeks after tumor inoculation), and cytokines representing Th1 (TNF-α,) and Th2 (IL-10) were detected by ELISA. The pCEA-SARS-CoV group revealed significantly higher TNF-α and IL-10 production than the pCEA and control groups (*p *< 0.05) (Figure 2)

### Vaccination and therapeutic effects of orally-administered pCEA- and pCEA-SARS-CoV-transformed *S. typhimurium*

**Figure 3:**
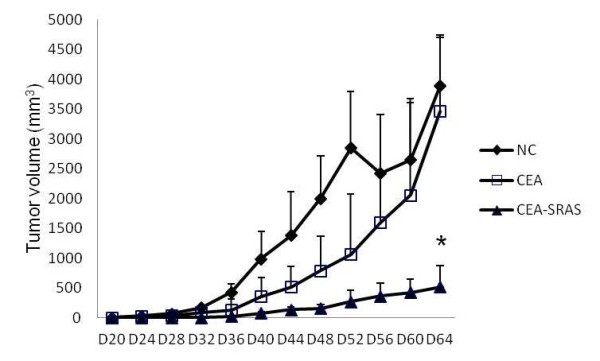
***In vivo *tumor development of BALB/c mice after oral vaccination**. Five BALB/c mice in each treatment group were inoculated with CT26/CEA cells 21 days after vaccination with pCEA- and pCEA-SARS-transformed *S. typhimurium *and the tumor volume in the mice was observed. (*, *p *< 0.05 in comparison with the control and the pCEA group).

To determine the efficacy of the pCEA-SARS-CoV tumor vaccine, mice were orally immunized with 1 × 10^8 ^pCEA- and pCEA-SARS-CoV-transformed *S. typhimurium *14 days before inoculation with colon cancer CT26/CEA cells. As per the data shown in Figure 3, the tumor volume was significantly lower in the pCEA-SARS-CoV group (*p *< 0.05) as compared with the pCEA group, indicating that the SARS fragment provided sufficient protection against the development of tumors.

**Figure 4:**
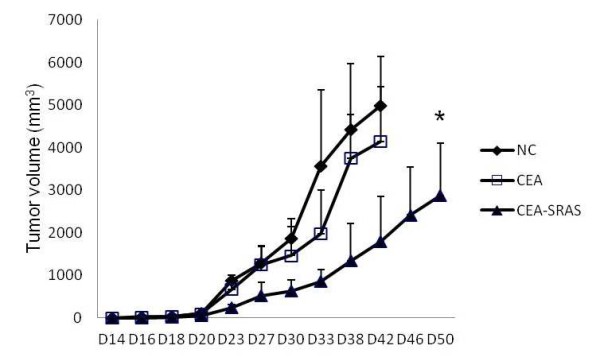
**Therapeutic effect of CEA-SARS-derived epitopes on tumor establishment**. BALB/c mice were inoculated with CT26/CEA cells on day 0. Four days later, the mice were fed with pCEA- and pCEA-SARS-transformed *S. typhimurium *once per week (5 mice/group), and the subsequent tumor volume in the mice was observed. (*, *p *< 0.05 in comparison with the control and the pCEA group).

Regarding the therapeutic effects of the vaccines, mice were inoculated with 1 × 10^5 ^CT26/CEA and 4 days later orally immunized with 1 × 10^8 ^*S. typhimurium *transformed with pCEA, or pCEA-SARS-CoV in 20 μl PBS, while the negative control group was fed with 20 μl PBS. While therapy with pCEA was not able to induce responses to reduce the growth of tumors in comparison with the control group, mice in the CEA-SARS-CoV group exhibited significantly less tumor growth, indicating that the SARS fragment provided adequate protection against CT26/CEA (Figure 4, *p *< 0.05 in comparison with the control and the pCEA group).

## Discussion

Our results demonstrated that fusion peptides composed of a highly-immunogenic epitope and a poorly-immunoreactive tumor-associated antigen can effectively activate host immunity, and furthermore, using the prediction algorithm SYFPEITHI to enhance the affinity of antigen presentation, the resulting fusion peptides provoked even stronger immune responses. In addition, this study also provided new insight into tumor-specific immunity. By combining SARS-CoV epitope, a tumor unrelated antigenic fragment, with CEA, we finally demonstrated this could be a promising anti-cancer strategy through effectively increasing both Th1 and Th2 cytokines and decreasing tumor volume.

The DNA vaccine, though usually injected intramuscularly, can be administered orally in an animal model with bacteria, and it has been observed that some display preferential replication or preferential accumulation in the tumor microenvironment [8]. Among all bacteria, *S. typhimurium *is one of the most utilized to carry therapeutic transgenes. Attenuation of *S. typhimurium *with partial gene modifications does not affect its ability to achieve high tumor/normal tissue ratios in mouse models, and the bacteria maintain their capacity to inhibit the growth of both subcutaneous tumors and lung metastatic diseases [8]. Other modifications have also been reported; for example, the strain used in this study was attenuated aromatic acid-dependent (*aro*) *S. typhimurium*, which has been well-characterized as a carrier of various heterogeneous antigens [9,10]. These vaccine strains are capable of colonizing the gut-associated lymphoid tissues (Peyer's patches) and secondary lymphatic tissues including the spleen and liver following oral administration in mice, eliciting mucosal, humoral and cell-mediated immune responses [11]. Previous studies have proven the lipopolysaccharides on the *Salmonella *surface enable CD14+ monocytes to secrete TNF-α, IL-1 and IL-6 [12,13]. Also *S. Typhimurium *could deliver directly into the cytosol of macrophages and dendritic cells resulting in vigorous antigen-specific CD8+ T cell priming and the induction of protective immunity against viruses, bacteria and tumors [14,15].

Variations in the cytokine profiles arose after vaccination with pCEA-SARS-CoV. As a highly-immunogenic peptide, treatment with SARS-containing epitopes has elicited a striking increase in TNF-α expression as compared with the negative control and the CEA group (Figure 2a). TNF-α is a proinflammatory and Th1 cytokine, and as indicated by Austin and colleagues, TNF-α, IFN-γ, and IL-2 can be used to define cytotoxic T lymphocytes and Th1 effector populations [16]. Reducing local expression of TNF-α was found to be associated with B16-F10 melanoma outgrowth in mice while s.c. administrating TNF-α significantly suppressed primary tumor growth of melanoma [17]. IL-10, a typical Th2 cytokine, was enhanced in the pCEA-SARS-CoV group as compared with the negative control and the CEA group (Figure 2b). Though most often known as a Th2 cytokine, IL-10 is actually a pleiotropic cytokine with anti-inflammatory, immunosuppressive, and immunostimulatory properties [18], and exerts immunostimulatory effects on B cells, cytotoxic T-cell development and thymocytes [19]. As indicated by Wogensen et al., expression of an IL-10 transgene by insulin-producing pancreatic cells led to the accelerated onset of diabetes in NOD mice [20,21], with no inhibition of immune-mediated destruction of islets [22].

In the study of Moritani et al., NOD (Non-obese diabetic) mice expressing an IL-10 transgene in glucagon-producing pancreatic cells also developed accelerated diabetes [23]. Consequently, the expression of IL-10 in our model may exert not only a Th2 effect but also a Th1 effect. In general, the CEA antigen only induces a Th2 response, whereas the addition of a SARS fragment helps to induce and enhance both Th1 and Th2 responses. Th1 is more famous for its antitumor effects, although Th2 responses have been reported in autologous tumors, T-cells from patients with indolent non-Hodgkin lymphomas frequently exhibiting an activated but apoptosis-prone phenotype [24]. It may seem that the Th2 response might downregulate the effect of a Th1 response or contribute less to the antitumor activity, but Th2-dominated antitumor immunity has also been observed in some models. As reported by Chu et al. [25], their DNA vaccine, which comprised a modified core peptide of mucin1 (PDTRP) and a GM-CSF coding sequence at the C-terminus, induced better protection against tumor challenge, and that protection was correlated with type 2 immune responses manifested by an increased IgG1 to IgG2a antibody ratio and greater induction of GATA-3 and IL-4 mRNA than of T-bet and IFN-γ mRNA in spleen cells of vaccinated mice.

The enhancement of Th1 and Th2 in the SARS-fragment-containing treatment groups indeed displayed better immunoactivity and protective effects in CT26/pCEA-bearing mice. In comparison with the negative control group, no significant differences in tumor volume were observed in mice immunized with CEA alone, while the tumor volume was found to be smaller in the pCEA-SARS-CoV group in the protection and therapy assays (Figure 3 &4). These results may indicate that enhancement of the affinity of antigen presentation by the prediction algorithm increased the immunogenicity of the CEA-SARS peptide.

## Conclusions

This study has demonstrated a potential strategy to overcome traditional obstacles in TAA-specific tumor therapy, and has shown that it is possible for foreign SARS fragments to improve the anti-tumor efficacy of tumor vaccines against endogenous tumor antigens. Application of these kinds of highly-immunogenic peptides and the enhancement of peptide affinity through prediction software could serve as a platform to elicit powerful host immunity and eliminate tumor growth.

## Methods

### Cell culture

All cell lines were obtained from the American Type Culture Collection (VA, USA). CT26 (mouse colon cell, ATCC: CRL-2638), PT67 (mouse retrovirus-packaging cell line, ATCC: CRL-12284), and BALB/3T3 (mouse embryo fibroblast, ATCC: CCL-163.2) were cultured in RPMI media supplemented with 2 mM L-glutamine, 50 μg/ml of Penicillin/Streptomycin (P/S) and 10% heat-inactivated fetal bovine serum (FBS) (Biological Industries, Haemek, Israel) at 37°C under 5% CO2. CT26/CEA cells were generated by transfecting retroviral vectors (pMSCV neo) carrying CEA DNA fragments (NCBI accession No. Z21818, nucleotide (nt.) CEA_10804~10938_, 132 bp). CT-26 transfectant cells (CT26/CEA) were obtained by G418 (Protech, Taiwan) selection and maintained in the RPMI growth medium described above.

### Prediction of H2-Kd-immunogenic amino-acid peptides

A fragment derived from the SARS coronavirus (SARS-CoV) (NCBI accession No. AB263618) that has proved to be effective in eliciting IFN-γ secretion in humans [6] was selected for use in this study and fused with CEA, a low-immunogenic peptide for peptide-binding prediction. The SYFPEITHI scoring system [26] was then utilized to predict the affinity of CEA and CEA-SARS-CoV peptides towards binding to BALB/c H2-Kd molecules.

### Construction of vectors for CEA and CEA-SARS-CoV gene expression

The eukaryotic expression plasmid pAAV-MCS was chosen for gene insertion and expression under the control of a CMV promoter. Gene fragments containing CEA and SARS-CoV were both artificially synthesized by polymerase chain reaction (PCR) using a series of sense and antisense primers designed precisely according to the above two gene sequences (Table 1), which provided suitable restriction enzyme sites. Because the antisense sequences overlapped with the sense sequences for 20 bps in the designed primers, they will base-pair with each other. The CEA fragment with the restriction enzyme sites *EcoRI *5' and *XbaI *3' and pAAV-MCS were both digested with *EcoRI *and *XbaI *and then ligated to form pCEA. The SARS fragment with *XbaI *5' and *HindIII *3' and pCEA were both digested with *XbaI *and *HindIII *and then ligated to form pCEA-SARS-CoV.

### Oral infection of BALB/c mice with *Salmonella*

Plasmids were electroporated into the *Salmonella *vaccine vector (*S. typhimurium *strain SL3261) at 2.5 mF, 2.5 kV, and 200Ω for 4~5 msec, and quantification of the bacteria was performed by plating serial dilutions in LB plates. Six-eight-week-old female BALB/c mice were purchased from the National Laboratory Center (Taipei, Taiwan) and housed in a temperature- and light-controlled (12L:12D) environment at the Animal Maintenance Facility of National Chiao Tung University (NCTU) (HsinChu, Taiwan). The institutional animal care and use committee of NCTU approved this study before its start. Mice in the treatment groups were immunized with 1 × 10^8 ^attenuated *S. typhimurium *(in 20 μl PBS) transformed with the different vectors (pCEA and pCEA-SARS-CoV) three times in two weeks, while the negative control group was fed with 20 μl PBS alone.

### ELISA

To evaluate the cytokine profiles of the mice after immunization with pCEA- and pCEA-SARS-CoV-transformed *S. typhimurium*, sera samples were collected at day 14 and assayed for TNF-α and IL-10 expression by standard ELISA (R&D, MN, USA) in compliance with the manufacturer's instructions.

### Vaccine protection assay

For the vaccine protection assay, 6-8-week-old BALB/c mice were immunized using the protocol described in the previous section. One week after the last booster injection, mice were inoculated with 5 × 10^5 ^CT26/CEA cells and the tumor growth pattern was measured every 4 days. Tumor volume was calculated using the formula volume = length × width × height.

### Vaccine therapeutic assay

To evaluate the tumor therapy, BALB/c mice were inoculated with 1 × 10^5 ^CT26/CEA cells (in 200 μl PBS/mouse) and orally immunized with 1 × 10^8 ^*S. typhimurium *(in 20 μl PBS/mouse) 4 days later, followed by re-immunization once a week after the first injection.

### Statistical analysis

Results are expressed as mean ± SE. Student's *t *test was applied to compare the effects of treatment in the different groups, and the Mantel-Cox significance test was employed to analyze the survival rate. A value of *p *< 0.05 was considered significant.

## Competing interests

We (authors in this manuscript) declares that we have no significant competing financial, professional or personal interests that might have influenced the performance or presentation of the work described in this manuscript.

## Authors' contributions

KWL and CSL conceived the study aims and design, contributed to the systematic review and data extraction, performed the analysis and interpreted the results. SHK drafted the manuscript and performed the experiments. YCC, CHL, YTH, and SCY assisted in doing experiments. CJW and RPL contributed to the revision of the manuscript. All authors have read and approved the final version of this paper.
